# Construct validity in cross-cultural, developmental research: challenges and strategies for improvement

**DOI:** 10.1017/ehs.2025.3

**Published:** 2025-02-21

**Authors:** Nicole J. Wen, Dorsa Amir, Jennifer M. Clegg, Helen E. Davis, Natalia B. Dutra, Michelle A. Kline, Sheina Lew-Levy, Tanya MacGillivray, Gairan Pamei, Yitong Wang, Jing Xu, Bruce S. Rawlings

**Affiliations:** 1 Department of Psychology, Centre for Culture & Evolution, Brunel University London, London, UK; 2 Department of Psychology & Neuroscience, Duke University, Durham, NC, USA; 3 Department of Psychology, Texas State University, San Marcos, TX, USA; 4 School of Human Evolution and Social Change & the Institute of Human Origins, Arizona State University, Tempe, AZ, USA; 5 Graduate Program in Neurosciences and Behavior, Universidad Federal do Pará, Belém, Brazil; 6 Department of Psychology, Durham University, Durham, UK; 7 Department of Psychology, Simon Fraser University, Vancouver, BC, Canada; 8 Department of Psychology, The Chinese University of Hong Kong, Hong Kong, Hong Kong; 9 Department of Anthropology, University of Washington, Seattle, WA, USA

**Keywords:** construct validity, cross-cultural, community-engaged research, culture-specific training, methodology, children

## Abstract

The recent expansion of cross-cultural research in the social sciences has led to increased discourse on methodological issues involved when studying culturally diverse populations. However, discussions have largely overlooked the challenges of construct validity – ensuring instruments are measuring what they are intended to – in diverse cultural contexts, particularly in developmental research. We contend that cross-cultural developmental research poses distinct problems for ensuring high construct validity owing to the nuances of working with children, and that the standard approach of transporting protocols designed and validated in one population to another risks low construct validity. Drawing upon our own and others’ work, we highlight several challenges to construct validity in the field of cross-cultural developmental research, including (1) lack of cultural and contextual knowledge, (2) dissociating developmental and cultural theory and methods, (3) lack of causal frameworks, (4) superficial and short-term partnerships and collaborations, and (5) culturally inappropriate tools and tests. We provide guidelines for addressing these challenges, including (1) using ethnographic and observational approaches, (2) developing evidence-based causal frameworks, (3) conducting community-engaged and collaborative research, and (4) the application of culture-specific refinements and training. We discuss the need to balance methodological consistency with culture-specific refinements to improve construct validity in cross-cultural developmental research.

## 1. Introduction

Interest in cross-cultural research has grown recently (Adetula et al., 2022; Barrett, 2020; Schimmelpfennig et al., 2024). This expansion has led to increased attention and discourse on the challenges of conducting cross-cultural research, and in particular, *construct validity*, when studying culturally and geographically diverse populations. *Construct validity –* the assessment of how well a tool or instrument measures the underlying construct it is intended to measure (Amir & Bornstein, 2024) – cannot automatically be assumed to be high across diverse cultural contexts. Despite the importance of construct validity, it is often overlooked in psychological research (Chester & Lasko, 2020), and this is particularly pertinent in cross-cultural research (Burger et al., 2023). Many common experimental approaches have been designed and validated in subsamples of western populations by western researchers (Burger et al., 2023; Draper et al., 2023; Hruschka et al., 2018), and such research is often conducted in non-native languages (Blasi et al., 2022; Peña, 2007; Stibbard-Hawkes et al., 2024), introducing additional challenges to validity and interpretation. Many implicit and unchecked cultural assumptions are often embedded in these protocols, which can further undermine their validity.

However, much of the conversation remains focused on research with *adults*. It fails to take into account the unique considerations that arise relating to conducting cross-cultural research with children. While developmental research – research that primarily focuses on infants and children – remains critical for a complete understanding of human behaviour, it also presents unique challenges for construct validity and requires tailored solutions. In recent years, developmental researchers have called for the use of diverse samples (Nielsen et al., 2017; Singh et al., 2023), improved cross-cultural workflows (Burger et al., 2023), and better engagement with theoretically driven, ethical, equitable, and community-engaged research practices (Amir & McAuliffe, 2020; Broesch et al., 2020, 2023). A key component of these improvements is a serious consideration of construct validity in developmental research to ensure that measures accurately assess what researchers intend, across culturally diverse contexts. These concerns are well founded; recent studies suggest that widely used research practices in developmental research exhibit low construct validity across contexts (Holding et al., 2018; Lew-Levy et al., 2021; Zuilkowski et al., 2016). Children (and adults) from different cultural contexts bring different norms, perceptions, expectations, and ways of responding to any given situation, making it difficult to confidently assume that a test is measuring what it intends – particularly when tests are designed and validated in one cultural context but applied in another.

In this paper, we provide guidelines for addressing concerns related to construct validity in cross-cultural developmental research. We begin by defining construct validity and exploring how it is measured, as well as examining its relevance to common methods used in developmental research. We then discuss key challenges researchers face and propose potential solutions, using examples of ours and others’ successes and failures (for an overview, see Table 1). We focus on how researchers can strike an optimal balance between methodological consistency and culturally appropriate protocol refinement. In doing so, we aim to raise awareness of these issues and to continue to improve the scientific rigour of cross-cultural developmental research.

**Table 1.** Overview of challenges and solutions for maximizing construct validity in developmental cross-cultural research

Challenge	Solution
**Lack of cultural and contextual knowledge:** Cross-cultural researchers may lack deep familiarity with the cultural contexts they study, often rushing experimental work, which can result in poorly designed measures.	**Including ethnographic and observational approaches:** Collecting qualitative data is essential for gaining a deeper understanding of the study’s cultural context and for informing experimental design. Combining both quantitative and qualitative data enables researchers to identify data patterns and develop interpretations that are grounded in the cultural context.
**Dissociating developmental and cultural theory and methods**: Cross-cultural experiments are often designed to be ‘clean’, to examine social-cognitive abilities in isolation and separately from cultural influences.	**Theory-led research design:** Accurately understanding childhood requires careful consideration of cultural context when developing research projects. Research questions should be thoughtfully refined using developmental and cultural theory, drawing on both experimental and ethnographic literature.
**Lack of causal frameworks:** Cross-cultural research often lacks a formal causal framework and may not clearly define underlying assumptions when determining cause-and-effect relationships. This can reduce confidence in inferring causal links between variables.	**Employing evidence-based, flexible causal frameworks:** Using a formal causal framework enables researchers to accurately identify suitable study populations, select relevant variables (rather than capturing everything possible), and establish causal relationships, leading to more precise conclusions. Multisite studies should begin by clearly defining population differences based on specific study objectives or goals. Tools like Directed Acyclic Graphs (DAGs) are helpful for examining causal directionality.
**Superficial, short-term partnerships and collaborations:** Cross-cultural research requires deep, ethical, and equitable collaboration, yet many partnerships are short-term and vertical, and are often dominated by western researchers. This dynamic can result in western theories and methods going unchallenged, even when they fall short in explaining observed phenomena.	**Conducting community-engaged and collaborative research.** Community-engaged and participatory research yields more accurate and ecologically valid results. Ideally, community members should be involved throughout the entire research process, from research design and framing research questions to data interpretation and sharing findings. However, meaningful engagement can also occur at any single stage. Collaboration with local stakeholders is especially crucial for enhancing construct validity in cross- cultural research, as it brings in essential cultural knowledge, disciplinary expertise, and insights that improve methodological development, research implementation, and dissemination.
**Culturally inappropriate tools and tests**: Cross- cultural research often involves transferring protocols designed and validated in one cultural context (usually western) to other populations, without systematically and rigorously checking their construct validity across these different contexts.	**Culture-appropriate measures**: Protocols must be culturally appropriate with maximal construct validity. They should be translated, developed, and rigorously piloted, with careful interpretation of pilot data. The protocols should be administered to ensure samples perceive and respond as intended. Regular validity checks should be incorporated throughout the research process. Input from local researchers and stakeholders familiar with the cultural context is crucial to ensure protocols are not unduly influenced by the implicit biases of researchers from different cultures. Adequate time should be allocated in projects for these important steps.

### 1.1. What is construct validity?

In its simplest form, construct validity is the extent to which an instrument or test measures the concept it is designed to (Borsboom et al., 2004; Stone, 2019). Measures with high construct validity should correlate strongly with other variables theoretically related to the target construct (Campbell & Fiske, 1959; Dahl, 2017). For example, a measure of children’s *theory of mind* – the ability to reason that humans have their own mental experiences (Fabricius et al., 2021) – with good construct validity should strongly correlate with other instruments designed to test theory of mind. The assessment of construct validity has a long history in psychological research (Strauss & Smith, 2009) and its applications continue to be debated today (Chester & Lasko, 2020; Stone, 2019).

Scientific testing is itself a cultural practice, as are the cognitive and behavioural measures commonly used in developmental science (Ardila, 2005; Greenfield et al., 1997). The scientific method, as a practice, was historically developed in the context of western societies, and therefore its approach is imbued with western values such as those of universalism, individualism, objectivity, neutrality, and quantification (Chaudhary & Sriram, 2020; Greenfield et al., 1997). However, the scientific method was influenced by a wide range of cultural ideas, practices, and institutions that had evolved over vast periods and across different regions, and which had come from various societies: this included input from non-western cultures, such as those in Africa, the Arab world, India, and China.

Methodologists have extensively explored the concept of construct validity and its connections to other forms of validity, such as internal and external validity (e.g., Campbell, 1957; Cronbach & Meehl, 1955). They have proposed various methods for evaluating and improving construct validity, including approaches like convergent and discriminant validity and the multitrait-multimethod matrix. This discourse extends to disciplines such as education (Kane, 2016; Messick, 1989), epidemiology (Matthay & Glymour, 2020), and social and personality psychology (Flake et al., 2017; Rohrer, 2024). The concept of ‘measurement invariance’ addresses whether a task or questionnaire is interpreted consistently across different groups, and is used to ensure it measures the same latent construct. Building on the proposal to treat the operationalization of psychological constructs as an optimisation problem (Moreau & Wiebels, 2022), we argue that prioritising construct validity is essential for rigorous and reliable research.

Despite early concerns in developmental research regarding measurement invariance across settings, culture included, the fact that any cognitive test is culturally laden has historically been downplayed, overlooked, or ignored across the mainstream literature (Ardila, 2017; Gould, 1981; Greenfield et al., 1997). Applying core concepts from measurement theory and psychometrics to cross-cultural research is critical. There has been progress in addressing construct validity across cultures in adult research – particularly with the adaptation and translation of psychological tests for diverse educational contexts (Ercikan et al., 2023). However, several biases still find their way into cross-cultural research, and special considerations must be taken into account for studies with children. In short, cross-cultural research is at a point now at which we can, and should, learn from existing research practices to improve future ones.

### 1.2. What do we know about construct validity in cross-cultural research?

As multisite and international research projects have proliferated, discourse on best practices for cross-cultural research has largely, though not exclusively, focused on work with adults, particularly when it comes to the validity of experimental protocols and methodologies. Hruschka et al. (2018) argue that many protocols employed by social and behavioural scientists were developed by and for a culturally unique subset of humans – typically those from affluent western populations – and were further refined with this population in mind. This can be problematic in cross-cultural research, because tools developed as reliable measures for one particular cultural context may translate poorly to others (Hruschka et al., 2018; Zuilkowski et al., 2016). We often underestimate the extent to which individuals are embedded in culture in our everyday experience. As Kluckhohnn (Kluckhohn, 1949, p. 11) stated, ‘It would hardly be fish who discovered the existence of water.’ Like humans embedded in culture, fish are surrounded by and immersed in water and therefore are unlikely to notice it. Hruschka et al. (2018) discuss their own failures in exporting a psychological protocol (social discounting) developed with western, highly educated adult populations to other populations. This protocol failed to measure social discounting in a rural Bangladeshi sample, because these participants had different frameworks for social closeness that were not captured by this western-derived measure. Additionally, participants needed concrete rather than hypothetical representations of their choices. This showcases potential problems with researchers’ unchecked assumptions about the universality of abstract concepts and decision-making processes. In describing the process of adapting their social discounting protocol, Hruschka and colleagues emphasised the need for the systematic adaptation and refinement of research methods using input from local stakeholders to account for cultural differences before conducting studies.

Other researchers have echoed the call to improve construct validity when working with populations distinct from those where measures were developed. Spake et al. (2024) addressed the challenge of balancing instrument standardisation with cultural sensitivity in cross-cultural work. They made clear recommendations for ensuring the validity of protocols, including (1) pre-registration, (2) early input and involvement of local collaborators, (3) thorough piloting to validate instruments, and (4) creating detailed standardised operating procedures to maintain consistency and reliability across diverse study settings. Further, Burger et al. (2023) emphasise that ensuring the validity of protocols in cross-cultural research is one of the most crucial, yet often one of the most neglected necessities in the field. They propose the creation of clear and systematic protocol development workflows that have formal validity checks embedded in them and the use of contextual qualitative information to improve ecological validity. Sanches de Oliveira and Baggs (2023) argue that theoretical motivations and methodological assumptions in the field of psychology are deeply entrenched in WEIRD (western, educated, industrialized, rich, and democratic; Henrich et al., 2010) biases. Using intelligence testing as an example of culturally biased assessments that were developed to sample specific skills relevant to western settings, the authors highlight the importance of validating assessments and measures in different contexts. Finally, Pamei et al. (2023) examined the construct validity of international literacy measures and highlight the importance of considering the specificity of scripts and languages when assessing and interpreting reading proficiency. These insights and recommendations all share a focus on taking a more nuanced and culturally informed approach to developing research protocols across diverse cultural contexts.

### 1.3. Discussion of construct validity should be extended to developmental work

While much of the conversation and most of the recommendations presented above regarding construct validity in adult work can be extended to developmental research, the latter presents unique theoretical and methodological challenges requiring specific consideration. Humans are strongly shaped by their sociocultural environment and the relationship networks in which they grow (Bronfenbrenner, 1979; Greenfield et al., 2003).

Developmental researchers aim to understand the extent to which developmental processes are shared or differ across populations, as well as the underlying mechanisms behind these similarities and differences (Broesch et al., 2023). Answering such questions is not easy, and requires the use of carefully designed protocols and sensitive ethical procedures as well as an understanding of developmental and cultural theory. Through the lens of construct validity, children vary across cultural contexts in terms of the norms surrounding and their levels of familiarity with the conditions in which data (particularly experimental data) are typically collected. These varying norms can include those related to being recruited and observed by unfamiliar adults, deference to authority or adults (Xu, 2019), performing unfamiliar tasks under scrutiny, following direct instructions, or using more formal or unfamiliar language than is typical (Rogoff, 2003). Because children are in a continuous state of physical, cognitive, and social growth, their developmental constructs are inherently more dynamic than those in adults. For instance, cognitive processes like attention or memory evolve significantly with age, so a test valid for one developmental stage may be unsuitable for another. Research with children involves stricter ethical guidelines and practical considerations than that involving adults. These considerations include children’s shorter attention spans, the need for frequent breaks, and requirements for parental consent. These constraints limit the scope and robustness of the methods, posing additional challenges to achieving construct validity.

Designing culturally grounded and valid measures is critical in developmental research, and previous work has shown that failing to do so can lead to biased results. For example, Lew-Levy et al. (2021) found that only 2% of Congolese BaYaka forager children and 14% of Bandongo fisher–farmer children, both in the age groups 4–12 years, solved a measure of tool innovation that required reshaping a pipe cleaner into a hook to retrieve an out-of-reach reward (called the *hook task*). These rates were *far* lower than those of their western counterparts (typically 50% by 7–8 years) – the context in which the task had been designed and validated (Rawlings, 2022). A superficial interpretation of these results may lead to the inference that BaYaka and Bandongo children are simply *worse* at tool innovation than western children. However, the authors also conducted naturalistic observations of how these children interacted with the pipe cleaners outside of the experimental setting. Here, the authors noted impressive innovation, with children using the pipe cleaners to make jewellery, decorations, and play items. This suggests that the hook task had low construct validity and was an inappropriate measure of tool innovation for this population. Without the accompanying observational data, these results could have led to misleading or potentially damaging conclusions. Similarly, Zuilkowski et al. (2016) examined pattern reasoning in children in rural Zambia using western-derived two-dimensional stimuli. The Zambian children identified patterns at lower rates than western norms until the task was redesigned into a 3D version (using beans and stones), after which performance improved. The authors suggested that Zambian children’s lower level of exposure to 2D materials (e.g., books, screens) compared to their western peers made the 2D version of the task an invalid measure of pattern recognition in this context.

In cross-cultural developmental research, it is common to ‘export’ protocols developed outside the cultural contexts in which they are being used – a practice that many of the authors of this manuscript have also engaged in. We recognise the ways in which the biases and assumptions this may bring can be harmful, both ethically and scientifically (Broesch et al., 2020). Our goal is not to finger-wag but rather to identify and discuss problematic practices, examine their implications, and advocate for positive, field-wide change. We hope to make a strong case for researchers to develop methods and practices that are culturally and contextually sensitive, and that are tailored to construct validity.

## 2. Challenges to construct validity in developmental cross-cultural work

### 2.1. Lack of cultural and contextual knowledge

Cross-cultural research has often relied on the ‘universality assumption’ (Kline et al., 2018), the belief that a lack of behavioural variation across cultural contexts necessarily means that a theory or construct is universal. Conversely, the presence of behavioural variation is considered evidence of cultural variation. However, for theories and constructs to be truly universal, their meaning must be consistent across cultures, and culture itself is the ultimate source of meaning (Pepitone & Triandis, 1987). For example, men in Fiji and Scotland both wear skirt-like garments on the lower halves of their bodies as part of their formal wear, but the superficial similarities of these outfits do not signify a shared cultural meaning or heritage. Similarity alone does not equate to cultural equivalence. On the other hand, the church and state organizations of Fiji and Scotland differ from each other along many dimensions, despite sharing a recent history of English colonial interference disrupting formerly autonomous rule. This example illustrates how over-reliance on surface similarities, without interrogating the deeper origins of cultural norms, can lead to misinterpretations of cross-cultural similarities and differences. This is exacerbated by the tendency of cross-cultural developmental science to overlook the critical insights provided by ethnography and observation, which can offer an in-depth understanding of cultural contexts and how we should study constructs.

Ethnographic approaches involve collecting in-depth qualitative data through interviews, surveys, and observations to better understand the cultural context in which behaviours develop and occur. These observations provide valuable cultural insights into children’s development. Constructs such as parenting styles, goals, or cognitive processes may hold different meanings across cultures, potentially leading to misunderstandings or misinterpretations of data if these are not examined and understood within their cultural contexts. For example, Keller et al. (2006) found that while certain western cultures (e.g., Germany, the USA, and Greece) emphasise independence in child-rearing, non-western cultures (e.g., rural Cameroon and rural India) prioritise interdependence and social cohesion, highlighting the importance of cultural context in understanding developmental behaviours. Without context, researchers might interpret the lack of independent behaviours in children in non-western cultures as a sign of delay or dependency, but they are in fact in line with cultural values in the population. By integrating ethnographic methods and gaining personal experience within the community of interest, researchers can better develop experimental protocols and interpret results. This approach allows for a more nuanced understanding of development across diverse cultural settings and enhances the validity of experimental measures.

Working with a community to better understand how and why children respond to experimental protocols can provide a richer understanding of constructs of interest. For example, in addition to the earlier examples of innovation and pattern recognition, the value of community input was clearly demonstrated in a cross-cultural study of mirror self-recognition (MSR) in young children (Broesch et al., 2010). In the MSR task, children are surreptitiously marked on the face or body and then shown their reflection in a mirror. The underlying assumption is that if a child recognises the reflection as themselves, they will attempt to remove the mark upon discovery. The majority of children in Kenya did not pass the task in the classic sense (by removing the mark when faced with the mirror image); instead they ‘froze’ and did not respond to the protocol, which could have been misunderstood as their having ‘failed’ the task. However, through discussions with local adults, researchers identified that the children’s freezing behaviour could be understood as hesitation about removing the mark. The children were acutely aware that the experimenter had intentionally placed the mark on them and did not want to disobey an authority figure. This is a poignant example of how a result that initially did not align with expectations revealed the importance and value of community input and contextual understanding in interpreting behaviour.

There are also cases where ethnographic follow-up could have significantly enriched our understanding of children’s development. For example, in a pilot study of an economic game where children could either share or keep sweets for themselves (Kline, 2008, field notes), researchers observed that children in a Yasawa (Fiji) village frequently collected large amounts of sweets for themselves during the game. In many contexts, this would have been interpreted as a self-serving behaviour. However, the researchers also noted that immediately after the game, the children distributed the sweets to other children, kin, and neighbouring households (Kline, 2008, field notes). Similarly, Wiessner (2009) found that Ju/’hoan Bushmen were more selfish in a dictator game and an ultimatum game because the anonymity of the games removed cultural institutions that governed sharing and reciprocity behaviour. These observations suggest that a sole interpretation based on the participants’ responses to the experimental protocol does not fully capture the meaning of their sharing behaviour. Including observational measures as part of the study design could offer a more accurate understanding of what the experimental data reveals within its broader cultural context.

Developmental scientists aim to extract meaningful insights from the historical, social, and ecological contexts in which development occurs, where complex interactions between these levels impacts development. This task becomes even more challenging when considering developmental changes, as children, their social partners, and their interactions are in a constant state of flux. The challenge is further compounded for researchers with limited experience or knowledge of the specific region or culture they are studying. Numerous assumptions shape the development of a research programme, questions, and constructs, and researchers may not always be aware of these biases – such as by providing a task that is over-reliant on 2D materials or interpreting ‘freezing’ as non-responsiveness. For developmental scientists conducting cross-cultural research, it is crucial to identify and avoid these biases and assumptions. Developing culturally grounded and valid measures requires careful planning, systematic checking, refinement, and thoughtful implementation – a process that often contrasts with the fast-paced nature of academic research (Rafiq et al., 2024). Unfortunately, the academic environment today often prioritises speed and quantity over depth and quality, fostering competitive, high-pressure research environments (Fernandez-Cano, 2021; Frith, 2020). Unreflective application of western-constructed and validated protocols to non-western cultures can lead to difficulties in inference and interpretation (Kline et al., 2018).

### 2.2. Dissociating developmental and cultural theory and methods

Contextual theoretical approaches, such as cultural-historical psychology, bioecological theory, and mediational theories of mind (Bronfenbrenner, 1979; Cole, 1996; Rogoff, 2003), emphasise the importance of examining the child at various contextual levels. It is surprising that we continue to run experiments and develop measures with only an individual child’s response in mind. Children do not, and cannot, develop in isolation and, like others in the field, we suggest that if we are to have an accurate understanding of childhood, we must take time to consider the cultural context when developing research projects.

Despite a history of research showing that nature and nurture are intertwined, many well-intentioned researchers (ourselves included) have tended to approach culture as a thing to remove, isolate, control for, or work around. The goal is often to develop ‘clean’ tasks and protocols to examine social-cognitive abilities as isolated from cultural influences. Sometimes these tasks are developed to be performed with just one individual child (see Broesch et al., 2010; Callaghan et al., 2005), and sometimes dyadically (see Corbit et al., 2021), with a caregiver (see Clegg et al., 2021), or in a group. These tasks may then be performed by a local individual with the objective of removing any barriers to understanding the task (e.g. language – verbal and non-verbal), or social barriers (e.g. performing a task with a stranger). It is possible that with the use of this widely accepted approach, we are only removing a language barrier (Stibbard-Hawkes et al., 2024). For instance, in our research in Vanuatu (Smit et al., 2019), although we asked a local adult to be the experimenter for a task, this did not guarantee that the researchers had a fluid comprehension of the language spoken by the local experimenter and the child. In one case, it took a week to accurately translate seemingly straightforward questions about sleep practices (e.g. ‘What time do you go to sleep most nights?’). Issues like these could potentially be avoided with clearer communication, but power dynamics and other motivations often prevent smooth and clear communication about such concerns (Urassa et al., 2021). Language barriers specific to a particular construct are just one of the many cultural layers that may unknowingly influence our results. One needs only to consider a contextual model to construe the endless possible influences that lead to individual and group decision-making.

Epistemology and methodology are deeply intertwined. Mainstream developmental psychology has failed to describe and understand children’s development in non-western populations (Nielsen et al., 2017; Sanches de Oliveira & Baggs, 2023). In fact, western psychology has a long history of describing development as a series of universal processes within an individual child that is independent of context.

Developmental theories are based on epistemological assumptions that guide the way we conduct research and interpret the behaviours we study. However, these underlying assumptions are often unexamined or unrecognised. Many methods used in developmental psychology are based on western standards, making them inadequate for capturing developmental processes even in contexts in which one is familiar (see Dahl, 2017). Developmental science operates within a broad set of concepts, making assumptions about the developing child and their developmental context, forming the conceptual framework that guides day-to-day research activities. For example, we assume that the child is born into a context in which they have parents and siblings who will scaffold the child for success in formal educational settings.

It is essential to articulate and examine the presuppositions or philosophical assumptions upon which theories, research, and methodologies are based. For instance, Stanton (2013) pointed out that Indigenous epistemologies are very different from those valued by mainstream society. Specifically, Indigenous communities believe that experiences are best shared in a dynamic, interactive, and face-to-face context; however, mainstream academic epistemologies value professional contexts that use written forms. Moreover, Indigenous epistemologies value subjectivity and multiple perspectives (Guimarães, 2020), which contrasts with the academic focus on reliability, validity, and consistency. A move away from Eurocentric epistemology would contribute to the development of methodologies that are more relevant to the cultural-specific contexts and would enhance construct validity in cross-cultural research.

### 2.3. Lack of causal frameworks

Though cross-cultural developmental research has emphasised experimental and reductionist methods, along with standardised protocols, it often fails to adopt robust causal frameworks. Most research questions in the behavioural sciences are causal in nature (Potochnik, 2017; Rohrer, 2024). There has been a recent increase in calls for more rigorous consideration of causal assumptions (the underlying assumptions when determining cause-and-effect relationships) and analyses (McElreath, 2022), including in cross-cultural research (Deffner et al., 2022), to improve study validity. Causal analysis can benefit from integrating both quantitative and qualitative methods (Blersch et al., 2022). We encourage researchers to adopt a formal causal inference approach throughout the research process, from study design and participant recruitment (Greenland, 2022) to data analysis and interpretation (McElreath, 2022).

Recently, researchers have argued that cross-cultural scientists need to carefully consider a formalised causal inference approach to dealing with key steps in the research process (Deffner et al., 2022; McElreath, 2022). By ‘formalised’ we mean explicitly identifying the effect of interest (often referred to as the theoretical estimand) (Lundberg et al., 2021) and the empirical estimand (the data generated by specific tasks) (Chatton & Rohrer, 2024), and describing the relationship between the two using Directed Acyclic Graphs (DAGs). This allows us to pre-emptively address the assumptions for potential claims of validity (Rohrer, 2024). In simple terms, a formalised causal inference approach makes clear what effect we are trying to examine and under what conditions we can confidently claim to have found an effect. While many studies may claim to have found a relationship between two variables, without a formal causal inference framework, we cannot be certain that the observed effect is genuine (Rohrer, 2024).

To illustrate, consider intelligence as it is commonly operationalized and measured using non-verbal intelligence tasks. A more precise theoretical estimand of intelligence can be stated as the ‘expected score of a child in the Raven’s Progressive Matrices task, averaged over all school-going children in rural Thailand and Laos’. Using this approach allows us to circumvent the limitations of interpreting models (here, for intelligence) which are poorly formalised (van Hoogdalem & Bosman, 2023), with vast variations of empirical evidence across different contexts. Following Deffner et al. (2022), we argue that a formalised framework can be a valuable tool to help describe and approach concerns in a more systematic way.

### 2.4. Superficial, short-term partnerships and collaborations

Cross-cultural research almost always requires extensive and multifaceted collaboration. Increasingly, publications and guidelines are emphasising the importance of effective, ethical, and equitable partnerships in developmental cross-cultural research (Broesch et al., 2020, 2023; Burger et al., 2023; Spake et al., 2024; Urassa et al., 2021). However, many cross-cultural collaborations are short term, reflecting the traditional experimental model followed by mainstream psychology. Within these teams, collaborations are often vertical, with western researchers dominating discussions and leading projects, often in their native languages. Psychology researchers from non-western regions frequently report experiencing bias from their western counterparts, particularly when they conduct research in their own regions or are affiliated with non-western institutions (Raval et al., 2023). These dynamics contribute to the lack of construct validity in cross-cultural research, as western theories and methods remain unchallenged, even when they fail to adequately explain observed phenomena. Our goal is to approach collaboration development through the lens of construct validity, ensuring that all perspectives are equally valued and incorporated.

Optimal collaboration is particularly important for improving construct validity in cross-cultural research because it introduces expertise in terms of cultural knowledge, academic disciplines, methodological development, and the implementation and dissemination of research to diverse networks. For example, input from researchers and stakeholders from the study of cultural context can help develop protocols that are perceived and administered as intended, reducing the influence of implicit biases from researchers from different cultural contexts (Hruschka et al., 2018). Contribution from various disciplines can provide complementary perspectives or approaches. Researchers using experimental methods in cross-cultural research can gain valuable insights from anthropologists, who use qualitative methods to deepen their understanding of the study population, and vice versa (Weisman & Luhrmann, 2020).

### 2.5. Culturally inappropriate tools and tests

Improving the validity and rigour of any study depends on a well-designed research plan and proper training of the research team, particularly those administering and coding tasks. This is especially important in international and cross-cultural developmental research, which often involves diverse groups of researchers across different geographical locations. In such studies, research questions must be carefully refined using developmental and cultural theory. Additionally, protocols must be translated, developed, rigorously piloted (with pilot data carefully interpreted), and administered in a way that ensures participants perceive them and respond as intended.

Translated materials must be appropriate and accessible for all intended recipients, including participants, community members, stakeholders, and research assistants. Ensuring this accessibility should be an integral part of the research process. This is particularly critical when working with children, as complex instructions or language can easily be misunderstood. One common cause of errors in cross-cultural research is poorly translated materials. To avoid misinterpretation, it is essential that words and phrases accurately capture the intended constructs; this requires the investment of careful attention and time. This diligence ensures that any variation in performance reflects true cultural differences, rather than issues with construct validity (Amir & McAuliffe, 2020; Burger et al., 2023; Holding et al., 2018; Pamei et al., 2023). This concern differs from protocols that are simply poorly worded; the prevalence of English in academia can introduce systematic bias, especially when research begins with English-language materials that are shaped by American or British cultural assumptions.

In many studies, children are rewarded for participation, with common rewards in a western context including stickers, sweets, toys, and sometimes small amounts of money in exchange for participation. Rewards and payments are not always appropriate across different developmental populations due to contextual variations. In Brazil, for example, monetary rewards are forbidden in any kind of scientific research by the ethical legislation, and other types of reward are assessed on a case-by-case basis. Meanwhile in the USA, monetary rewards to children are relatively common. Researchers can either standardise or match rewards across populations or offer culturally specific ones, which can mean providing markedly different rewards for participants in different cultural contexts.

Providing both the same and different rewards across diverse populations can evoke varying levels of motivation or different perceptions, leading to disparities in engagement and outcomes of the research. In some cultures, monetary rewards of varying amounts might be highly motivating, while in others, social recognition or communal benefits are more effective. Additionally, the same reward may elicit different levels of motivation across populations of children, (e.g., sweets in one culture may not be as novel and motivating as in another, where they are not commonly found). Ignoring these differences can lead to unintended consequences, such as demotivation or misinterpretation of the rewards’ value, ultimately compromising the validity and reliability of the findings.

A high-profile example of this is the marshmallow task (Mischel et al., 1988), which measures delayed gratification by testing whether children will wait to eat one marshmallow for the promise of a second marshmallow. Willingness to wait has been associated with better outcomes later in school, relationships, and health (Michaelson & Munakata, 2020). One study found that Japanese children delayed gratification for a marshmallow longer than American children. Yet when the reward was changed to a wrapped gift, American children delayed gratification longer than Japanese children (Yanaoka et al., 2022). Waiting to eat is emphasised more in Japan than it is in the USA, whereas waiting to open gifts is emphasised more in the USA than it is in Japan. This highlights the importance of considering the cultural context for different rewards and incentives in study design to increase construct validity.

## 3. Potential solutions for issues relating to construct validity in developmental cross-cultural work

We now offer a set of solutions to address the challenges identified above. These solutions are not exhaustive but are based on our own experiences as well as the theoretical and empirical literature.

### 3.1. Including ethnographic and observational approaches

To enhance the construct validity of our experimental protocols, we recommend combining experimental methods with ethnographic and observational approaches. Collecting both quantitative and qualitative data enables researchers to identify patterns and develop contextually grounded interpretations (Dahl, 2017; Rogoff et al., 2018; Xu, 2017). We suggest three possible approaches for designing experiments that integrate both observational and ethnographic data.

1. The first approach involves using an experimental protocol followed by observations. This method helps the researcher to understand cultural pathways within universal developmental processes (Greenfield et al., 2003). Experimental results can facilitate direct cross-cultural comparisons, allowing researchers to assess whether a phenomenon occurs similarly across cultures. This approach offers a way to compare performance in a controlled way. However, on its own, it may not capture cultural dynamics fully. While experimental results can confirm or reject a hypothesis, it may not be clear how meaningful a behaviour observed in an experiment is within a specific cultural context. Therefore, we recommend pairing experiments with observational and interview data to provide more meaningful insights.

As Kline et al. (2018) suggest, it is possible to observe the same behaviour in different societies, yet the underlying mechanisms driving those behaviours may differ, or different behaviours in separate populations may stem from the same underlying mechanism. This principle is central to evolutionary anthropology, which seeks to explain how one ‘human nature’ can result in diverse cultural practices. An example is the ‘polygyny threshold’ model, which assumes that specific ecological conditions make polygyny more likely than monogamy (see Ross et al., 2018 for a review and critique). Similarly, in developmental research, ‘statistical learning’ of language follows a comparable approach – one set of rules for all developing children can lead to highly diverse outcomes, depending on the cultural context. Because developmental processes and outcomes are not 1:1 pairs, it is crucial to investigate both processes and outcomes across different measures before drawing developmental conclusions from cross-cultural comparisons.

1. A second approach involves conducting an experiment to challenge or confirm ecological validity. If an experiment is not yielding the expected results, researchers can pair those findings with observations and interviews to demonstrate that the experiment is not accurately capturing the intended construct. Lew-Levy et al. (2021) applied this approach when examining tool innovation using the hook task with Congolese BaYaka forager children and Bondongo fisher–farmer children. They used structured observations and interviews to explain why their experimental findings did not support their predictions in terms of a methodological mismatch.
2. The third approach involves starting with observations (either ethnographic or quantitative) and subsequently develop hypotheses and design a culturally specific experiment to test the dynamics observed. This approach is particularly useful for deepening cultural understanding or ‘keeping the tension’ between fieldwork and ethnography (Astuti, 2017). Experiments in this approach are effective for eliciting implicit knowledge that may not be easily verbalized (e.g., sharing norms) or that may be too complex for young children to verbalize. Lew-Levy et al. (2022) employed this approach to examine the developmental trajectory of intra- and interethnic sharing in the Republic of Congo, using a dictator game and basing predictions about the point at which these sharing norms would stabilise on ethnographic insights. While observational and ethnographic data can be time-intensive, integrating them into the research design helps prevent researchers from engaging in ‘helicopter research’, where they remain detached from the community.

### 3.2. Employing evidence-based, flexible causal frameworks

When adopting a formal causal inference approach, we suggest that cross-cultural science can learn from efforts made in other fields, such as health research (Haber et al., 2022), sociology (Lundberg et al., 2021), and personality psychology (Rohrer, 2024). Drawing on Deffner et al. (2022), we present a brief set of guidelines for applying a causal framework to cultural developmental research.

Multisite studies should begin by clearly defining how the populations differ based on the specific study objectives or goals. The first step is to clarify what the theoretical estimand is, which is the key focus when conducting empirical analysis. The theoretical estimand is the unit-specific quantity for each target population. For example, we might estimate that the probability of 5–10-year-old children solving a task in a given population is 60%. Once the theoretical estimand is set, we need to attach it to the empirical estimand, which allows us to estimate the probability that all 5–10-year-old children in the study population will solve the task based on data collected from our sample (since we typically cannot collect data from every individual in a population).

Tools like DAGs are helpful to linking theoretical and empirical estimands (for an introduction and application to DAGs, see Rohrer, 2018; Wysocki et al., 2022). DAGs are a graphical and mathematical tool to help researchers identify a priori causal assumptions between variables with three major structures – a confounder (a variable that independently influences both the predictor and outcome variables, X <- Cf -> Y), a mediator (a variable *on* the predictor–outcome causal path that influences the outcome variable, X -> M -> Y), and a collider (a variable mutually affected by both the predictor and the outcome, X -> Co <- Y). DAGs also contain nodes which capture whether the populations or contexts of interest differ from each other in relevant aspects. In the previous example of intelligence, a selection node ***S*** in a DAG can be used to represent the assumption that regions differ in their school starting age. If we want to study the effect of schooling on intelligence then we can have a variable, school starting age (instead of current age), as a selection node. Thinking ahead about such potential nodes facilitates the principled selection of variables for cross-cultural studies. Thus, DAGs help researchers understand and explain the data, as well as identifying the potential presence of selection bias (Hernán et al., 2004). Since cultures differ across many dimensions, using a formal causal framework enables researchers to identify appropriate study populations, select specific variables to measure (rather than capturing everything possible), and pinpoint causal relationships, leading to more accurate conclusions (Deffner et al., 2022).

We also recognise the tension between proposing technical (statistical) solutions and advocating for an inclusive approach to cross-cultural research. Complex statistical techniques often require tools and training that are inaccessible to many researchers worldwide. As the expectation of sophisticated statistical approaches in research grows, many departments in wealthy, western institutions (in disciplines such as psychology or anthropology) have begun hiring dedicated statisticians or offering consultancy services to assist researchers. This problem is further exacerbated by the spread of generative-AI models (e.g., large language models) which often require exceptional amounts of computational resources. However, many researchers may not have access to training, appropriate software, or statistical support. These disparities can create inequities in publishing opportunities, as researchers from wealthier institutions often have an advantage when it comes to submitting to mainstream journals. This highlights a significant issue of academic inequality that we must continue to acknowledge and address (IJzerman et al., 2021).

We note, however, that implementing a causal approach doesn’t necessarily require advanced training or resources. The first step is careful, transparent thinking about which variables influence the outcome measure. Descriptive information of relevant environmental and cultural variables, alongside rich ethnographic information, is critical to strong causal frameworks with clearly defined study populations. In cases where complex statistical and causal approaches are unavailable – or even when they are – we encourage researchers to include a ‘Constraint on Generalisability Statement’. This statement should explicitly identify and justify the target populations in empirical papers (Simons et al., 2017; Tiokhin et al., 2019). The inclusion of this statement will afford more accurate inference and improve study replicability.

### 3.3. Conducting community-engaged and collaborative research

#### Community-engaged research

The importance of including community members in cross-cultural research, particularly in developmental science, is gaining increasing attention (Oppong, 2022; Rad et al., 2018). Many studies are still conducted without the involvement of Indigenous people, perpetuating colonial hierarchies in decision-making processes (Napoli, 2019). However, there is growing recognition of the need to rethink research practices in ethical and culturally appropriate ways, particularly in sociocultural contexts which some members of the research team may be unfamiliar with. A community-engaged approach, which emphasises collaboration between researchers and participants, has emerged as an alternative that fosters research inclusion and engagement with multicultural populations (Rodriguez Espinosa & Verney, 2021). Specifically, community-engaged research involves community members equitably throughout the entire research process, aiming to uphold principles of participation, cooperation, collaboration, empowerment, and knowledge translation (Mathie & Cunningham, 2003). This approach creates opportunities to build meaningful partnerships with communities. The goal of involving communities is not only to develop scientifically valid research methods but also to ensure that the research is relevant and beneficial to the target community (den Houting et al., 2021). This is essential for conducting both accurate and ethical science, particularly when studying children, as caregivers are best positioned to advise on children’s behaviours and contextualize our observations and findings.

Ideally, community members should be involved in all stages of the research process, from the design and framing of research questions to data interpretation and knowledge dissemination. However, community engagement can occur at any stage of the research (see Wang et al., in prep.). Community-engaged and participatory research typically yields more accurate and ecologically valid results (Hruschka et al., 2018; Quintanilha et al., 2015; Urassa et al., 2021). Even as local researchers, it is crucial to recognise that different communities hold unique perspectives shaped by factors such as history, race, religion, and socio-economic status. These factors influence power dynamics and privilege within research collaborations, including with Indigenous communities, school communities, low-income groups, LGBTQ + populations, and immigrant communities (Urassa et al., 2021). When working with vulnerable communities, researchers should focus on community strengths and assets rather than taking a deficit-based approach (Suarez-Balcazar et al., 2022).

To improve research with communities and enhance the translation of research findings, it is crucial to provide authentic and consistent support to communities on their own terms (Oppong, 2023). This approach not only offers researchers more opportunities to assess the validity and rigour of their measures but also facilitates the practical application of research findings (Mathie & Cunningham, 2003; Wang et al., in prep.). One successful example of integrating community perspectives with scientific methods comes from environmental science research in Atlantic Canada, where Indigenous communities have adopted the ‘two-eye-seeing’ approach. This approach, coined by Elder Albert Marshall and practiced by Dr Bartlett, combines Indigenous local knowledge with western scientific perspectives to offer a more holistic and rigorous understanding of science (Bartlett et al., 2012; Marshall et al., 2015). We encourage developmental cross-cultural researchers to adopt a similar integrative approach.

#### Collaborative research

While some researchers have extensive networks of collaborators, many do not, and building such networks to conduct valid research is a complex challenge. There is no one-size-fits-all approach for establishing international and cross-expertise collaborations. Researchers come from diverse backgrounds, across disciplines, with varying levels of experience, networks, and methodological approaches. For example, someone at an institution with a strong focus on developmental cross-cultural research will likely have a different starting point than someone at an institution with little interest in this area. Similarly, wealthy (often western) institutions often have more resources and opportunities for building collaborations than those with fewer resources. Although the literature provides some general recommendations for establishing collaborations, these were not specifically designed for cross-cultural (developmental) research, although they still offer valuable guidance. A key theme in these recommendations is the importance of opening communication channels early, setting clear expectations and goals, addressing ethical and logistical challenges, and considering potential conflicts of interest (Croghan et al., 2022; Gjerde et al., 2024; de Grijs, 2015). We support these recommendations and strongly encourage prioritising equitable collaborations (Urassa et al., 2021).

The literature often lacks specific guidance on *how* researchers should contact potential collaborators. This may be due to the significant differences in levels of access, networks, and experiences among global researchers, which make it difficult to provide broad recommendations. One direct way to identify and engage potential collaborators is through discussions at conferences. However, many researchers face barriers to attending international conferences, which are often held in expensive western cities, making it difficult for researchers from low- or middle-income countries to participate. Additionally, visa restrictions may also pose challenges. While hybrid conference formats have improved accessibility to some extent, issues such as registration fees, time-zone differences, and limited informal networking opportunities remain. Moreover, internet access for streaming and virtual meetings can still be unreliable.

A more straightforward approach is to directly contact researchers working in the relevant field or location. Platforms are available to support international collaboration. For example, the Psychological Science Accelerator connects nearly 2,500 researchers across 73 countries (as of this writing) who collaborate on multisite research projects, allowing researchers to join projects that interest them. Similar networks exist in other fields, such as the Collaborative Anthropology Network for anthropology, as well as topic-specific platforms researchers can engage with.

Once collaborations are established, it’s crucial to focus on enhancing the validity of research protocols. We recommend fostering an open environment where all team members are receptive to input from the beginning, allowing protocols to evolve and be shaped by experts familiar with the cultural context of the study. Regular communication is essential, and setting up multiple communication channels – such as email, virtual meetings, and messaging apps – can help accommodate differences in accessibility and time zones.

### 3.4. Culture-specific refinement and training

Culturally appropriate research design and thorough training of the research team are essential for enhancing validity and rigour in cross-cultural studies. Many principal investigators (PIs) and labs already have effective workflows for training and research design, with published guidelines available to help improve these methods (Burger et al., 2023; Spake et al., 2024). Our aim here is to offer additional suggestions that can be integrated into these workflows specifically to strengthen construct validity.

Whether a protocol is newly developed or has been adapted from an established one in another cultural context, it is essential for collaborators to prioritise input from local community members (Burger et al., 2023). Protocols should be piloted extensively whenever possible, with pilot data shared with the collaborative team for review and refinement as needed. If translations are required, team members should ensure a rigorous process, such as using a multi-step translation and back-translation approach (Burger et al., 2023; Holding et al., 2018; Ozolins et al., 2020). This cycle should be repeated until all parties are confident that the protocols are accurately understood as intended. The same careful attention should be given to disseminating findings, ensuring that published conclusions are culturally appropriate and informed by all collaborators to prevent misunderstandings or potentially harmful interpretations.

We emphasise that collecting pilot data is a critical part of research design and protocol development, requiring careful and systematic planning. Pilot studies – small-scale versions of the main study – can reveal potential strengths and weaknesses in protocols, highlight risks of failure in full-scale studies, and indicate whether instruments are eliciting the intended responses from participants (van Teijlingen & Hundley, 2002). Developmental research involves complexities that demand careful interpretation and handling.

Where possible, we recommend that PIs collect pilot data themselves to gain first-hand experience with the protocols and to understand participants’ perceptions and responses prior to full-scale data collection. Although the extent of PI involvement in data collection varies by institution, career stage, and study type, this hands-on step allows project leaders to appreciate key study details, such as task duration, participant reactions, adherence to protocol, and potential improvements for validity. If direct involvement is not feasible, PIs can review video recordings of the pilot data collection to gain similar insights.

Additionally, we suggest creating clear, accessible training videos for research assistants that supplement written protocol instructions. These videos should be in the appropriate language and provide visual guidelines on protocol administration, allowing assistants to watch them repeatedly for clarity and confidence (Heller Murray, 2024). Training videos can be especially useful for complex studies or for assistants with limited data-collection experience. Research assistants should also be encouraged to provide feedback on the training materials to refine and improve them. In previous cross-cultural studies, some authors of this manuscript have further required research assistants to record themselves administering tasks and receive feedback in order to minimize experimenter error.

Additionally, the research team should design reward systems that are perceived as fair and equitable across different populations. While the specific form of the reward may vary (e.g., monetary rewards, food, etc.), its value and impact should remain consistent. It is essential to use culturally appropriate incentives to ensure ethical and effective participation. The rewards system should reflect and respect the diverse values, norms, and socio-economic contexts of participants, and should be informed by community input and piloting. To assess the appropriateness of incentives and their effect on participant motivation, researchers should test different types of incentives during the pilot phase. This will allow them to identify potential issues with inappropriate incentives early on. By tailoring rewards to align with cultural expectations and values, and ensuring they are seen as fair and respectful, researchers can build trust, encourage meaningful participation, and maintain ethical standards, all while enhancing the validity and reliability of the data.

All these steps require significant time, and researchers should account for this in their project timeline. Rushing into data collection risks outcomes such as poorly designed protocols, low construct validity, and study failures. A key benefit of developing detailed, well-written protocols, easy-to-follow training videos, and conducting extensive piloting is that it supports the open science movement by increasing study replicability and transparency. Clear guidelines and transparent criteria for reward distribution are also crucial for maintaining equity in cross-cultural developmental research. By establishing these guidelines, researchers can ensure that the reward distribution process is well documented and consistently applied across diverse cultural contexts. This documentation ensures fairness and consistency, while also providing a clear record of the criteria used to justify specific incentives, ultimately supporting research integrity and validity.

## 4. Conclusion

Nearly 15 years ago, researchers highlighted the significant over-reliance on wealthy western populations in the behavioural and social sciences (Henrich et al., 2010). Since then, efforts to diversify study samples have grown, leading to a notable increase in cross-cultural studies, including in developmental research. However, a common practice remains: researchers often apply measures developed for one population to another and make comparisons based on performance. We, along with others, argue that this approach presents several challenges to construct validity. In this article, we identify these challenges and provide guidelines to help researchers improve construct validity in cross- cultural developmental research.

Overcoming these challenges is not easy and requires careful, systematic planning. Researchers must engage with relevant developmental and theoretical frameworks, assemble a team of experts familiar with both the discipline and cultural context of study, conduct thorough validity checks across the project, and, when possible, incorporate ethnographic data alongside formal causal frameworks to guide study design and analysis. All of this takes significant time, which is often a limited resource for researchers but must be accounted for in project planning. Researchers must also adapt to unforeseen challenges in how participants perceive and respond to protocols, and be aware that, when working in cultural contexts different from one’s own, implicit biases can affect methodological validity. They should be prepared for protocols to fail and use (and document) these experiences to improve construct validity for future work, both for themselves and others. We emphasise that protocols with high construct validity not only enhance the accuracy of researchers’ measures but help prevent incorrect and potentially harmful conclusions when comparing developmental populations.

## Research transparency and reproducibility interest

This manuscript does not rely on any data, code, or other resources.
